# GSK-3β and MMP-9 Cooperate in the Control of Dendritic Spine Morphology

**DOI:** 10.1007/s12035-015-9625-0

**Published:** 2016-01-06

**Authors:** Ilona Kondratiuk, Szymon Łęski, Małgorzata Urbańska, Przemysław Biecek, Herman Devijver, Benoit Lechat, Fred Van Leuven, Leszek Kaczmarek, Tomasz Jaworski

**Affiliations:** 1 Laboratory of Neurobiology, The Nencki Institute of Experimental Biology, 3 Pasteur, 02-093 Warsaw, Poland; 2 Laboratory of Neuroinformatics, The Nencki Institute of Experimental Biology, Warsaw, Poland; 3 Laboratory of Molecular and Cellular Neurobiology, The International Institute of Molecular and Cell Biology, Warsaw, Poland; 4 Faculty of Mathematics, Informatics, and Mechanics, University of Warsaw, Warsaw, Poland; 5 Department of Human Genetics, Experimental Genetics Group - LEGTEGG, KULeuven, Leuven, Belgium

**Keywords:** GSK-3β, MMP-9, Imaging, Transgenic mice, Dendritic spines, Synaptic plasticity

## Abstract

Changes in the morphology of dendritic spines are prominent during learning and in different neurological and neuropsychiatric diseases, including those in which glycogen synthase kinase-3β (GSK-3β) has been implicated. Despite much evidence of the involvement of GSK-3β in functional synaptic plasticity, it is unclear how GSK-3β controls structural synaptic plasticity (i.e., the number and shape of dendritic spines). In the present study, we used two mouse models overexpressing and lacking GSK-3β in neurons to investigate how GSK-3β affects the structural plasticity of dendritic spines. Following visualization of dendritic spines with DiI dye, we found that increasing GSK-3β activity increased the number of thin spines, whereas lacking GSK-3β increased the number of stubby spines in the dentate gyrus. Under conditions of neuronal excitation, increasing GSK-3β activity caused higher activity of extracellularly acting matrix metalloproteinase-9 (MMP-9), and MMP inhibition normalized thin spines in GSK-3β overexpressing mice. Administration of the nonspecific GSK-3β inhibitor lithium in animals with active MMP-9 and animals lacking MMP-9 revealed that GSK-3β and MMP-9 act in concert to control dendritic spine morphology. Altogether, our data demonstrate that the dysregulation of GSK-3β activity has dramatic consequences on dendritic spine morphology, implicating MMP-9 as a mediator of GSK-3β-induced synaptic alterations.

## Introduction

Dendritic spines comprise the postsynaptic compartments of excitatory inputs as the basic units of information processing and storage [1]. Dynamic changes in dendritic spine morphology, the growth of new spines, and the elimination of existing spines can occur on very different time scales, reflecting adjustments of the synaptic strength that supports learning and memory [2]. The regulation of dendritic spine morphology is governed by the extracellular matrix (ECM), cell adhesion molecules (CAMs), and the cytoskeleton that is controlled by specific signaling networks [3]. Components of the ECM, including matrix metalloproteinases (MMPs), have been proposed to actively contribute to dendritic spine remodeling. Accumulating evidence suggests a specific role for MMP-9 in regulating the structural plasticity of dendritic spines [4].

Glycogen synthase kinase-3 (GSK-3) is a serine/threonine protein kinase that besides glycogen metabolism also regulates many critical cellular processes in most organs, including the central nervous system [5]. GSK-3 exists as two isozymes, GSK-3α and GSK-3β, that have similar structures but are not functionally identical in neurons [6]. GSK-3β is the more essential isozyme, because its genetic deletion is lethal in mouse embryogenesis, in contrast to GSK-3α [7–11]. GSK-3β is ubiquitously present in the brain, including neurons and synaptosomes [12] that essentially represent excitatory synapses [13]. At excitatory synapses, GSK-3β balances two major forms of synaptic plasticity: long-term potentiation (LTP) and long-term depression (LTD), which are both *N*-methyl-d-aspartate (NMDA) receptor-dependent [14]. During LTP, NMDA receptor activation inhibits GSK-3β activity through phosphorylation at Ser9 via the PI3K/Akt pathway, whereas during LTD, GSK-3β activity increases [14]. This apparent duality in molecular mechanisms that require the modulation of GSK-3β phosphorylation at Ser9 during experimental LTP or LTD is crucial for learning and memory [12, 14, 15].

The dysregulation of signaling pathways that involve GSK-3 is associated with the pathogenesis of several neurological and psychiatric disorders, including mental retardation, schizophrenia, depression, and Alzheimer’s disease [16]. These disorders are also characterized by aberrant structural changes in dendritic spines.

Mouse models that lack or overexpress either of the GSK-3 isozymes mimic various pathological conditions that are observed in different neuropsychiatric and neurological disorders [17]. Balancing GSK-3 activity in mice by genetic or pharmacological manipulations can rescue some of the functional defects in behavior and synaptic transmission [12, 18–20]. Consequently, the pharmacological inhibition of GSK-3 was proposed as an attractive therapeutic strategy for mental illnesses. Psychoactive drugs (e.g., lithium salts) that are used to treat bipolar disorder, depression, and schizophrenia inhibit GSK-3, among other enzymes [21].

Despite growing evidence of the role of GSK-3 in functional synaptic plasticity [11, 12, 14, 15, 22–26], it is far from clear how GSK-3β controls structural synaptic plasticity, reflected by alterations in the number and shape of dendritic spines [27–29]. In the present study, we analyzed mice either overexpressing or lacking GSK-3β in neurons to clarify the relationship between aberrant GSK-3 activity and the structural plasticity of dendritic spines. We identified GSK-3β as a critical regulator of dendritic spine architecture. Unexpectedly, we also discovered that MMP-9, the extracellular MMP, acts as a downstream regulator of GSK-3β-induced dendritic spine alterations.

## Materials and Methods

### Animals

#### GSK-3β Transgenic and Knockout Mice

GSK-3β transgenic (TG; GSK-3β[S9A]) mice overexpress the constitutively active form of GSK-3β, with a mutation of Ser9 to alanine, specifically in neurons under the control of the mouse Thy-1 gene promoter. Heterozygous GSK-3β[S9A] mice were maintained on an FVB/N genetic background [30, 31]. GSK-3β[S9A] mice were compared with wild-type (WT) littermates as controls.

Neuron-specific GSK-3β-deficient (GSK-3β^n−/−^) mice were obtained by crossing mice with floxed GSK-3β genes with Thy-1-Cre recombinase transgenic mice. GSK-3β^n−/−^ mice were maintained on a mixed FVB-C57BL/6 genetic background [9]. GSK-3β^n−/−^ mice were compared with GSK-3β^loxP/loxP^ littermates, which lack Cre recombinase, as controls. For both genotypes, the Thy1-gene promoter has been shown to yield postnatal expression of the respective transgene in central neurons only [9, 11, 30, 31].

#### MMP-9 Transgenic and Knockout Animals

Transgenic Wistar rats overexpress autoactivating MMP-9 under the control of the synapsin 1 promoter (MMP-9 TG rats) [32]. MMP-9 homozygous knockout mice (MMP-9 KO mice) were obtained from Dr. Z. Werb (University of California, San Francisco) [33] and maintained on a C57Bl/6 background.

All of the animal experiments were performed by certified researchers in accordance with regional, national, and European regulations concerning animal welfare and animal experimentation. The researchers were authorized and supervised by the University Animal Welfare Commission (Ethische Commissie Dierenwelzijn, KULeuven, Leuven, Belgium) and the Ethical Committee on Animal Research of the Nencki Institute (Warsaw, Poland).

### Lithium Chloride Treatment

MMP-9 TG rats and MMP-9 KO mice were subjected to a regimen of 4 weeks of lithium salt treatment [34]. The experimental animals were divided into four groups (*n* = 3/group): WT animals that were fed a control diet (WT); KO and TG animals that were fed a control diet (MMP-9 KO, MMP-9 TG); WT animals that were fed a lithium salt-supplemented diet (WT + Li); and KO and TG animals that were fed a lithium salt-supplemented diet (MMP-9 KO + Li, MMP-9 TG + Li). Lithium chloride (0.2 % [*w*/*w*]) was supplemented in custom-prepared food pellets (Vivari, Warsaw, Poland). Treatment commenced at 3 months of age, and the diets were provided ad libitum for 1 month. Animals that fed the lithium salt-supplemented diet were given drinking water with 1.5 % (*w*/*v*) sodium chloride to counteract the peripheral side effects of lithium ions.

### Dendritic Spine Analysis

Dendritic spine analysis was performed essentially as described previously [35]. GSK-3β[S9A] and GSK-3β^n−/−^ mice and Li^+^-treated MMP-9 KO mice and MMP-9 TG rats and their respective controls were anesthetized with pentobarbital and transcardially perfused first with phosphate-buffered saline (PBS) and then with 1.5 % paraformaldehyde (PFA) at room temperature. The brains were postfixed in 1.5 % PFA for 20 min and transferred to ice-cold PBS for another 20 min. The brains were cut into 130-μm-thick slices using a vibratome and left in PBS for 1 h. The sections were labeled by gene gun delivery of tungsten particles (Bio-Rad, Hercules, CA, USA) coated with the lipophilic dye 1,1ʹ-dioctadecyl-3,3,3ʹ,3ʹ-tetramethylindocarbocyanine perchlorate (DiI; d-3911, Thermo Fisher). The slices were subsequently incubated in 1.5 % PFA for 24 h to allow the dye to diffuse into neuronal processes, including spines. Confocal images of secondary apical dendrites of the dentate gyrus field were acquired under 561-nm fluorescent illumination. Spines were measured and analyzed using semiautomatic custom software (SpineMagick) [36].

### Dendritic Spine Clustering

The virtual skeletons of dendritic spines were obtained in SpineMagick. Spine length was calculated as the length of the path from the spine top to the dendrite along the virtual skeleton of the spine. To analyze the shapes of spines, the virtual skeleton of each spine from an individual image was transformed to form a straight line. The images were then rescaled to normalize the spine area. For each spine diameter, we defined width as a function of distance from the dendrite, denoted d(h).

We classified 9429 spines according to shape from GSK-3β-modified mice and their respective controls using a two-step procedure [37]. First, all 9429 d(h) functions were clustered into 36 clusters. Second, the clusters were manually sorted into three groups (i.e., mushroom, stubby, and thin spines) based on visual inspection of clustered spines. The data analysis was performed using custom scripts that were written in Python using NumPy and SciPy [38, 39] and Matplotlib [40].

### Pentylenetetrazole Treatment

To induce seizures and activate MMP-9, the mice were injected with the γ-aminobutyric acid-A (GABA_A_) receptor antagonist pentylenetetrazole (PTZ) [41]. GSK-3β[S9A] (*n* = 6) and WT (*n* = 6) mice (3.5–4 months old) were habituated to handling by subjecting them to intraperitoneal (i.p.) injections of 0.9 % NaCl twice per day for 7 days before PTZ stimulation. On day 8, WT and GSK-3β[S9A] mice were divided into groups that received either PTZ (50 mg/kg, i.p.) or saline (0.9 % NaCl, i.p.). The mice were sacrificed by cervical dislocation 10 min after the onset of PTZ-induced seizures or after saline injection. This 10-min time point was chosen based on a timeline of MMP-9 activity upon PTZ treatment (50 mg/kg) in WT mice. The mice were sacrificed 0, 5, and 10 min after PTZ-induced seizure onset (*n* = 3/time point).

### Acute Hippocampal Slices and GM 6001 Treatment

GSK-3β[S9A] and WT mice (*n* = 5/group) were anesthetized with isoflurane and decapitated. The brains were quickly removed and placed in cold NMDG solution (135 mM *N*-methyl-d-glutamine, 1 mM KCl, 1.2 mM KH_2_PO_4_, 1.5 mM MgCl_2_, 0.5 mM CaCl_2_, 20 mM choline bicarbonate, and 10 mM d-glucose, pH 7.4) saturated with a carbogen gas mixture (95 % O_2_ and 5 % CO_2_). Both hemispheres were cut into 300-μm coronal slices with a vibratome. The slices were left for recovery in artificial cerebrospinal fluid (aCSF; 119 mM NaCl, 2.5 mM KCl, 26.2 mM NaHCO_3_, 1 mM NaH_2_PO_4_, 2.5 mM CaCl_2_, 1.3 mM MgCl_2_, and 10 mM d-glucose) and saturated with carbogen at room temperature for 1 h. Slices from WT and GSK-3β[S9A] mice were then transferred to aCSF that contained either 0.004 % dimethylsulfoxide (DMSO) or GM6001 (1 μM in DMSO) and incubated at room temperature for 1 h. Following fixation with 1.5 % PFA for 20 min, the slices were processed for DiI dendrite labeling and spine analysis.

### Western Blotting

Protein extracts were subjected to sodium dodecyl sulfate-polyacrylamide gel electrophoresis (SDS-PAGE; 8 % gels) and electrotransferred (semi-dry transfer) to polyvinylidene difluoride membranes (Immobilon-P, Millipore). The membranes were blocked with 10 % (*w*/*v*) dried nonfat milk powder in Tris-buffered saline with 0.1 % Tween-20 and incubated with the following primary antibodies: rabbit anti-pGSK-3α/β(Ser21/9) (1:1000 dilution; #9331, Cell Signaling Technology); rabbit anti-GSK-3α/β (1:1000 dilution; #5676P, Cell Signaling Technology); rabbit anti-pAktS473 (1:1000 dilution; #4060, Cell Signaling Technology), rabbit anti-pAktT308 (1:1000 dilution; #4056, Cell Signaling Technology); rabbit total Akt (1:1000 dilution; #9272, Cell Signaling Technology); mouse anti-β-dystroglycan (β-DG; 1:500 dilution; NCL-b-DG, Novocastra); and mouse anti-glyceraldehyde-3-phosphate dehydrogenase (GAPDH; 1:2000 dilution; MAB374, Chemicon). Following washing with TBST, the membranes were incubated with horseradish peroxidase-labeled secondary antibody (anti-mouse or anti-rabbit; Vector Laboratories). After washing, peroxidase activity was visualized with ECL plus reagent (GE Healthcare). Signal densities were analyzed using GeneTools software (SynGene, England).

### Matrix Metalloproteinase Gel Zymography

The extraction of MMP from mouse brain tissue was performed as described previously [42]. Following cervical dislocation, the brains were rapidly removed. The cerebral cortex was isolated, homogenized, and centrifuged. Proteins from the supernatant were precipitated with cold ethanol, and the precipitate was solubilized in sample buffer. The pellets from the first centrifugation (Triton X-100-insoluble) were resuspended, incubated at 60 °C, and centrifuged. The proteins from the resulting supernatant were precipitated and solubilized in non-reducing sample buffer.

Triton X-100-insoluble samples were subjected to SDS-PAGE on 8 % gels that contained 2 mg/ml gelatin (Sigma-Aldrich). Following protein separation, the gels were washed with 2.5 % Triton X-100 and incubated with moderate shaking in developing buffer for the enzymatic reaction. The gels were stained with 0.1 % Coomassie Blue G-250.

### Dissociated Hippocampal Cultures

Dissociated hippocampal cultures were prepared from newborn (postnatal day 0) Wistar rats [35]. The brains were removed, and hippocampi were isolated on ice in dissociation medium. The hippocampi were then dissociated with papain and rinsed in dissociation medium and MEM plating medium. The hippocampi were triturated in plating medium, and cells were diluted in OptiMEM (Thermo Fisher) and centrifuged. The cells were plated at a density of 120,000 cells per poly-l-lysine-coated coverslip (Sigma). The cells were kept in maintenance medium at 37 °C under a humidified 5 % CO_2_ atm. All of the experiments were performed on days 17–19 in vitro.

### Cell Stimulation

Hippocampal neurons were incubated for 5 min with 400 ng/ml of recombinant MMP-9 or inactive MMP-9 E402A mutant or with MMP-9 and wortmannin (100 nM in DMSO) in maintenance medium. The final concentration of DMSO did not exceed 0.016 %. After stimulation, the cells were washed with maintenance medium, lysed with reducing SDS sample buffer, and subjected to Western blotting.

### Statistical Analysis

Graphs were prepared using Prism 5.01 software (GraphPad, San Diego, CA, USA). Spine densities were compared using the Mann-Whitney test. The numbers of differently shaped spines were compared using the *χ* ^*2*^ test. The effects of lithium and the MMP-9 inhibitor crossed with the effects of GSK-3β modification in mice on dendritic spine morphology were statistically analyzed using nested Gaussian mixed models. Animals and photographs were considered nested random effects, whereas the inhibitor and genetic modification were considered crossed fixed effects. To stabilize variance, the length-to-width ratio was log-transformed. Modeling was performed using the R statistical package [43] with lme4 [44] (available on CRAN) and DendriticSpineR (available on GitHub). Graphs presenting densitometric quantification of WB were prepared in Excel and results were compared using Student *t* test.

## Results

### Mice Expressing Constitutively Active GSK-3β had Longer and Thinner Dendritic Spines, Whereas GSK-3β-Deficient Mice had Shorter Dendritic Spines

**Fig. 1:**
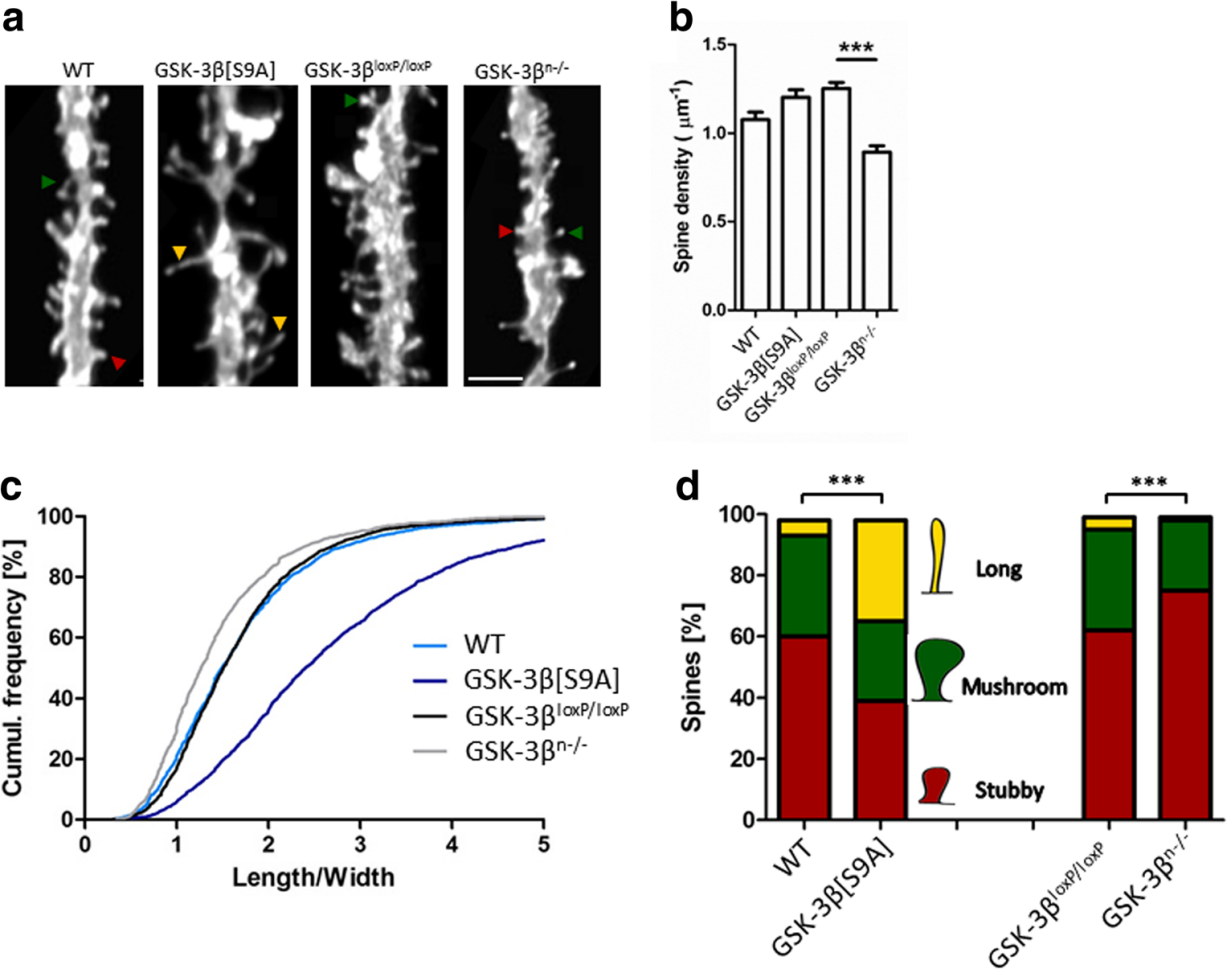
GSK-3β imbalance in neurons alters dendritic spine density and morphology. **a** Example photographs of DiI-stained secondary apical dendrites of granule neurons in the dentate gyrus in GSK-3β[S9A] and GSK-3β^n−/−^ mice. *Scale bar* = 2 μm. **b** Spine densities of dentate gyrus neurons in GSK-3β[S9A] and GSK-3β^n−/−^ mice. The data are expressed as mean ± SEM. ***p < 0.001 (Mann-Whitney test). **c** Cumulative histogram of dendritic spine length-to-width ratio in GSK-3β^n−/−^ and GSK-3β[S9A] mice (*p* < 0.001, vs. GSK-3β^loxP/loxP^ and WT; nested analysis of variance). **d** Spine morphology in GSK-3β[S9A] and GSK-3β^n−/−^ mice. ***p < 0.001 (*χ* ^*2*^ test). GSK-3β[S9A]: *n* = 6; WT: *n* = 6; GSK-3β^n−/−^: *n* = 4; GSK-3β^loxP/loxP^: *n* = 5 mice)

We studied how GSK3β affects structural synaptic plasticity in the adult central nervous system in mice either overexpressing constitutively active GSK-3β in neurons (GSK-3β[S9A]) or deficient in GSK-3β specifically in neurons (GSK-3β^n−/−^). We compared spine density and morphology with control WT mice and mice with a floxed GSK-3β gene (GSK-3β^loxP/loxP^), respectively. The morphometric analysis of spines in neurons that were stained with the DiI dye was performed in the dentate gyrus (Fig. 1a) using the length-to-width ratio as the most reliable reflection of spine morphology [35].

GSK-3β[S9A] mice had similar spine densities (Fig. 1b) but significantly longer spines, with a 60 % larger average length-to-width ratio than WT mice (Fig. 1c). Conversely, GSK-3β^n−/−^ mice had a 14 % smaller length-to-width ratio of dendritic spines compared with GSK-3β^loxP/loxP^ mice (Fig. 1c). Spine density was reduced by 28 % in neurons in GSK-3β^n−/−^ mice (Fig. 1b). Importantly, the two control strains (WT and GSK-3β^loxP/loxP^) did not differ with regard to the length-to-width ratio (Fig. 1c), although spine density in GSK-3β^loxP/loxP^ mice was 14 % higher than in WT mice (Fig. 1b).

To further understand how GSK-3β affects dendritic spine shape, we clustered spines into mushroom, stubby, and thin categories (Fig. 1d). GSK-3β[S9A] mice exhibited a significant increase in the population of thin spines (28 % more) and a significant decrease in the population of stubby spines (21 % less) compared with WT mice, whereas GSK-3β^n−/−^ mice exhibited an increase in the population of stubby spines (13 % more) compared with GSK-3β^loxP/loxP^ mice.

### GSK-3β Regulation of Dendritic Spine Morphology Involved MMP-9

The elongated phenotype of dendritic spines in GSK-3β[S9A] mice was reminiscent of a similar observation in MMP-9-overexpressing TG rats [35]. MMP-9 is an extracellularly acting protease that regulates dendritic spine shape [35, 45, 46]. Consequently, we hypothesized that GSK-3β regulates MMP-9 activity to control dendritic spine morphology. We further hypothesized that this co-regulation would be dysfunctional in the absence of GSK-3β (i.e., in GSK-3β^n−/−^ mice).

**Fig. 2:**
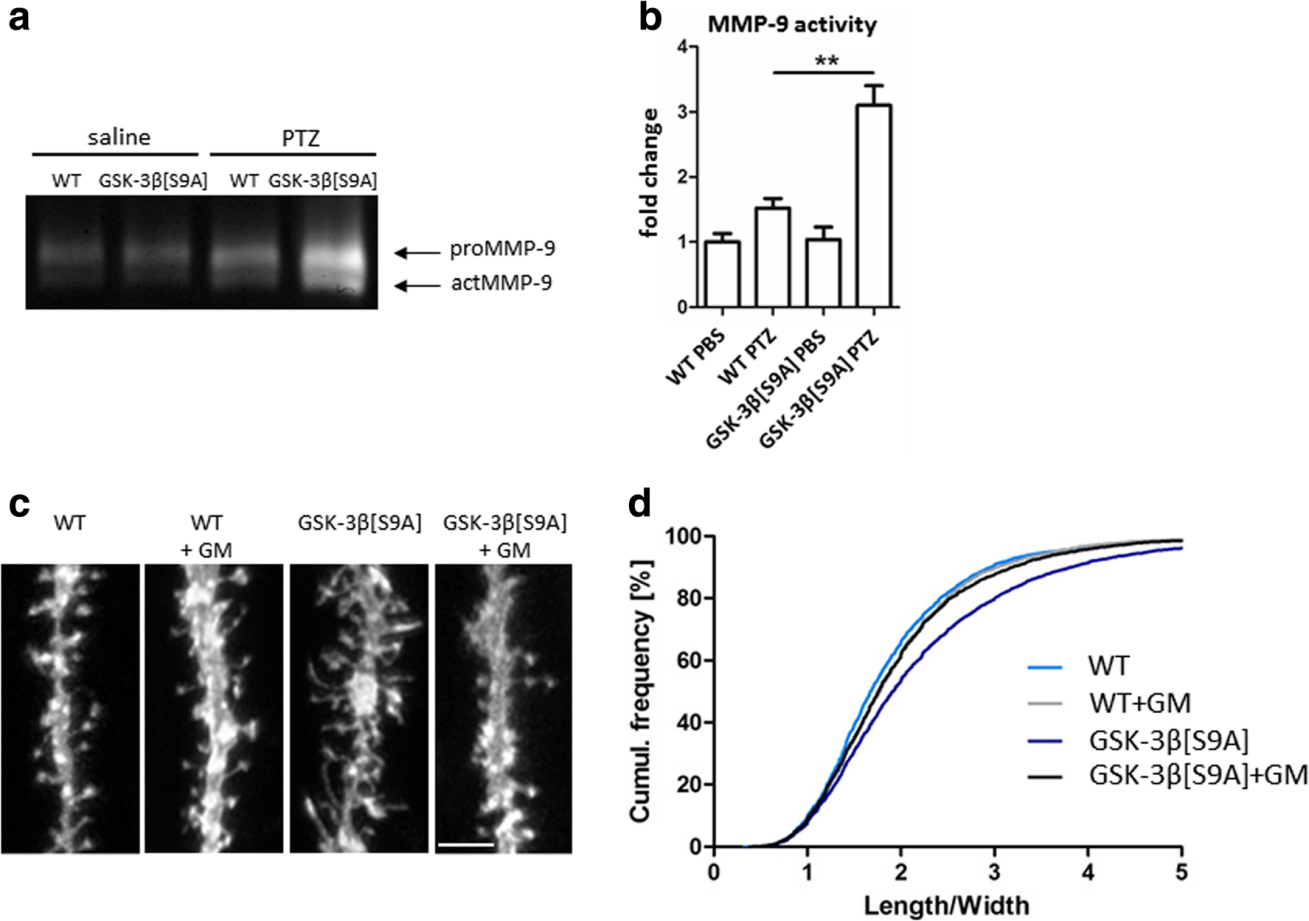
GSK-3β regulates dendritic spine morphology in an MMP-9-dependent manner. **a** WT and GSK-3β[S9A] mice were stimulated with saline or PTZ, and MMP-9 activity in hippocampal homogenates was analyzed by gelatin gel zymography. **b** MMP-9 activity in control and GSK-3β[S9A] mice. The data are expressed as mean ± SEM. **p < 0.01 (Mann-Whitney test). **c** Example photographs of DiI-stained apical dendrites of dentate gyrus neurons in acute hippocampal slices from WT and GSK-3β[S9A] mice after administration of the MMP-9 inhibitor GM6001. Scale bar = 2 μm. **d** Cumulative histogram of spine length-to-width ratio in WT and GSK-3β[S9A] mice (*p* < 0.01; nested analysis of variance). GM6001 normalized the dendritic spine length-to-width ratio in GSK-3β[S9A] mice (*p* < 0.01; nested analysis of variance). WT: *n* = 5; WT-GM6001: *n* = 5; GSK-3β[S9A]: *n* = 5; GSK-3β[S9A]-GM6001: *n* = 5 mice

To test these hypotheses, we analyzed MMP-9 activity in GSK-3β[S9A] and GSK-3β^n−/−^ mice (Fig. 2a). Because MMP-9 is released by synaptic stimulation, we habituated mice for 7 days to exclude any possible effects of the handling procedure. On day 8, the mice were injected with saline (basal condition) or PTZ to produce strong neuronal excitation. Previous studies reported that PTZ-induced MMP-9 activity in vivo, and the sensitivity to PTZ-induced epileptogenesis was MMP-9-dependent [32, 41]. Gelatin gel zymography demonstrated that MMP-9 activity was unaffected by GSK-3β[S9A] overexpression in the basal condition, whereas GSK-3β[S9A] potentiated MMP-9 activity almost twofold upon neuronal excitation (Fig. 2b).

Interestingly, these experiments showed that GSK-3β^n−/−^ mice were extremely susceptible to PTZ-induced seizures. On average, 50 % died within 3 min after the injection of 50 mg/kg PTZ. Lower PTZ doses (35 mg/kg) extended the survival of GSK-3β^n−/−^ mice to 10 min, but at this lower dose neither GSK-3β^loxP/loxP^ nor GSK-3β^n−/−^ mice developed seizures, with no changes in MMP-9 activity (data not shown). Consequently, the assessment of MMP-9 activity was not relevant in GSK-3β^n−/−^ mice.

We then sought to determine whether MMP-9 mediates GSK-3β-induced changes in dendritic spine morphology. We analyzed acute hippocampal slices from GSK-3β[S9A] mice, both without MMP inhibition and after MMP inhibition, and measured spines of granular neurons in the dentate gyrus (visualized by DiI dye; Fig. 2c). The length-to-width ratio was significantly higher (by 12 %) in GSK-3β[S9A] slices compared with WT slices, confirming the in vivo results (Figs. 1c, 2d). Interestingly, the application of GM6001 to GSK-3β[S9A] slices significantly reduced the length-to-width ratio by 10 % (Fig. 2d). These results indicate that increases in MMP-9 activity contribute to the elongation of dendritic spines under conditions of increased GSK-3β activity.

### Longer and Thinner Dendritic Spines Produced by Activated MMP-9 Were Normalized by Lithium Chloride

**Fig. 3:**
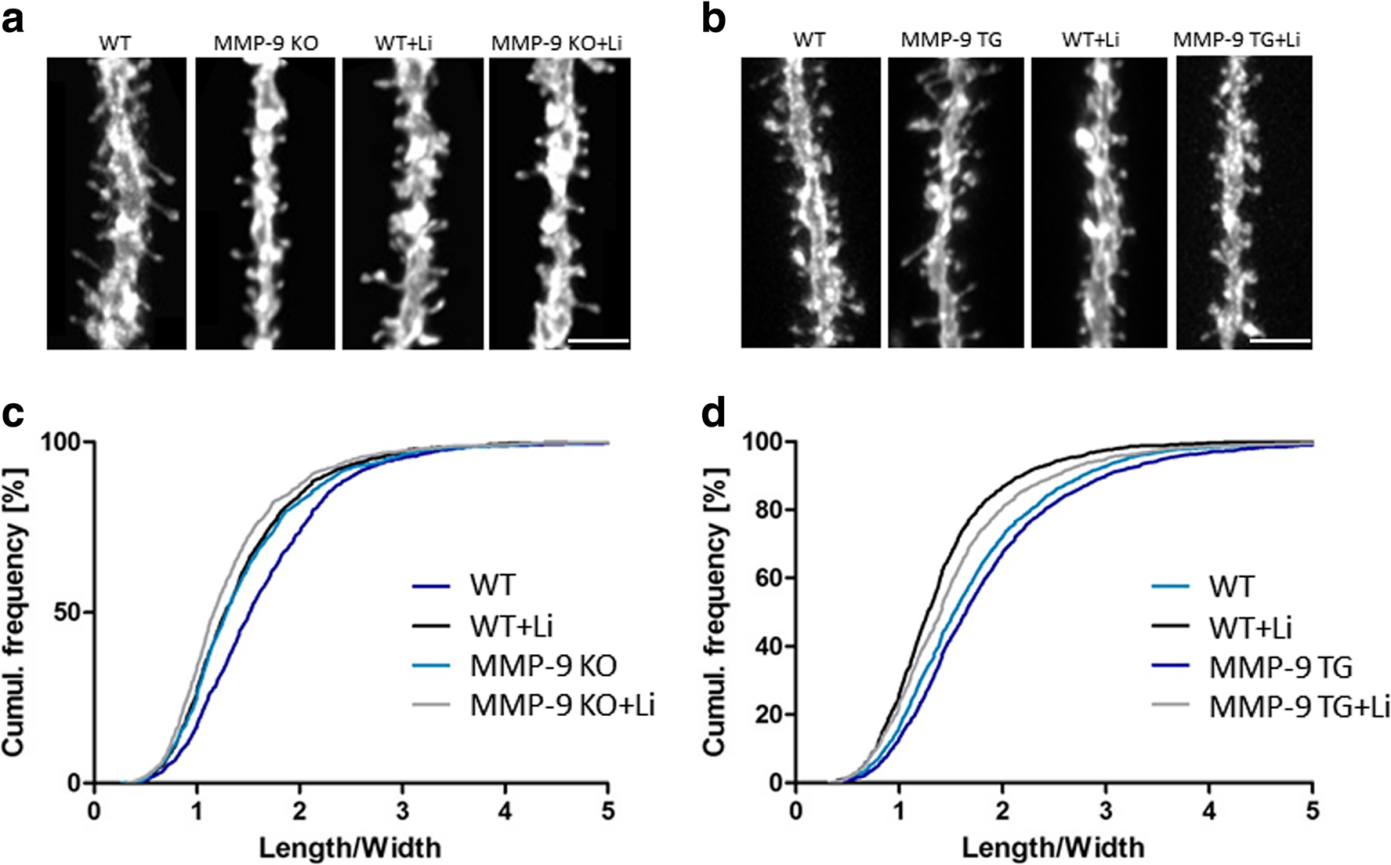
Dendritic spine morphology in MMP-9 KO mice and MMP-9 TG rats after chronic GSK-3 inhibition. **a** Example photographs of DiI-stained apical dendrites of dentate gyrus granule neurons in WT and KO MMP-9 mice treated with lithium chloride. **b** Example photographs of DiI-stained apical dendrites of dentate gyrus granule neurons in WT and TG MMP-9 rats treated with lithium chloride. *Scale bar* = 2 μm. **c** Cumulative histogram of dendritic spine length-to-width ratio in WT and KO MMP-9 mice treated with lithium. MMP-9 KO mice had shorter dendritic spines (*p* < 0.05; nested analysis of variance). Lithium significantly reduced the length-to-width ratio of dendritic spines in WT mice (*p* < 0.05; nested analysis of variance), with no effect in MMP-9 KO mice (*p* = 0.135; nested analysis of variance). MMP-9 KO: *n* = 3; WT: *n* = 3; WT-Li: *n* = 3; MMP-9 KO-Li: *n* = 3 mice. **d** Cumulative histogram of dendritic spine length-to-width ratio in MMP-9 TG rats. MMP-9 TG rats had a higher length-to-width ratio of dendritic spines (*p* < 0.05; nested analysis of variance). Lithium reduced the length-to-width ratio of dendritic spines in WT and MMP-9 TG rats (*p* < 0.001; nested analysis of variance). MMP-9 TG: *n* = 3; WT: *n* = 3; WT-Li: *n* = 3; MMP-9 TG-Li: *n* = 3 rats

To further investigate the effects of the relationship between MMP-9 and GSK-3β activity on the morphology of dendritic spines, we chronically inhibited GSK-3 in MMP-9 KO mice and MMP-9 TG rats. We used lithium because: (i) GSK-3 is inhibited by lithium ions in vivo [47–50], (ii) different and more specific GSK-3 inhibitors mimic the behavioral actions of lithium salt [51, 52], (iii) the deletion of GSK-3α or GSK-3β mimics the behavioral actions of lithium salt in mice [48, 53, 54], (iv) lithium salt-sensitive behavior is reversed by increasing brain GSK-3 activity [18], and (v) impairments in synaptic transmission caused by increased GSK-3β activity are normalized by lithium salt [12]. Animals were treated with lithium chloride for 30 days, and spines of neurons in the dentate gyrus were visualized using DiI dye (Fig. 3a, b ). MMP-9 KO mice had a significantly smaller (by 11 %) spine length-to-width ratio compared with WT mice (Fig. 3c ). MMP-9 TG rats had a significantly larger (by 8 %) length-to-width ratio compared with WT rats (Fig. 3d). Chronic lithium chloride treatment significantly reduced the spine length-to-width ratio in WT mice (Fig. 3c) and WT rats (Fig. 3d) by 13 and 18 %, respectively, producing a similar dendritic spine phenotype as neuronal GSK-3β deficiency (Figs. 1c, d and 3c, d).

If MMP-9 and GSK-3β regulate dendritic spine morphology through distinct molecular mechanisms, then the absence of MMP-9 activity should not affect the observed effects of GSK-3 on spine alterations. We observed no changes in the length-to-width ratio in MMP-9 KO mice that were treated with lithium chloride compared with untreated MMP-9 KO mice (Fig. 3c). In contrast, lithium chloride treatment reduced (by 14 %) the length-to-width ratio in MMP-9 TG rats (Fig. 3d). These results demonstrate that GSK-3 and MMP-9 act in concert on the same signaling pathway(s) to control dendritic spine morphology.

### MMP-9 Induced GSK-3β Phosphorylation Through the PI3K/Akt Signaling Pathway

**Fig. 4:**
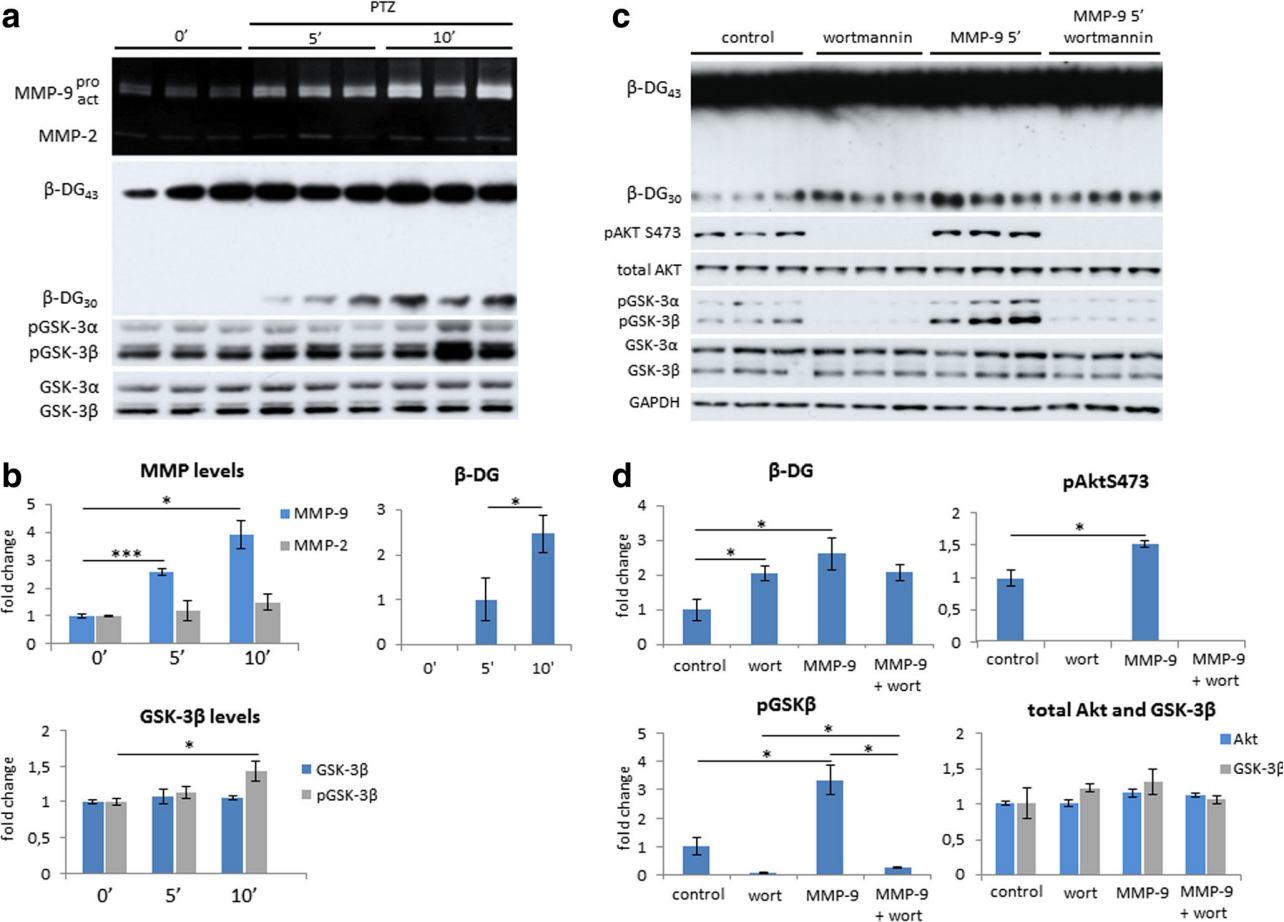
MMP-9 activity induced GSK-3β phosphorylation via PI3K/Akt signaling pathway. **a** Neuronal excitation induced MMP-9 activity, followed by GSK-3 phosphorylation, in PTZ-treated WT mice that were analyzed 0, 5, and 10 min after PTZ treatment. MMP-9 activity in total hippocampal homogenates was visualized by gelatin gel zymography (*upper panel*) and Western blot for β-DG cleavage (*middle panel*) and total and phosphorylated GSK3 isozymes (*lower panel*). **b** Densitometric quantification of pGSK-3βSer9, total GSK-β, β-DG and MMP-9 levels. The data are expressed as mean ± SEM. *p < 0.05 (Student *t* test). *n* = 3 mice for each condition. **c** Autoactive MMP-9 induced GSK-3β phosphorylation in hippocampal neuronal cultures. PI3K inhibitor wortmannin prevented MMP-9-induced GSK-3β phosphorylation. **d** Densitometric quantification of β-DG, pAktSer473, pGSK-3βSer9, total Akt, and total GSK-3β levels. The data are expressed as mean ± SEM. *p < 0.05 (Student *t* test). *n* = 3 culture wells for each condition

Higher GSK-3β activity concomitantly increased MMP-9 activity upon neuronal excitation. Therefore, we investigated whether secreted MMP-9 affects neuronal signaling. We analyzed MMP-9 activity in WT mice, in which MMP-9 was activated by an injection of PTZ (Fig. 4a). PTZ significantly increased MMP-9 activity, which coincided with increases in the levels of the cleaved 30-kDa form of β-DG, the neuronal substrate of MMP-9 (Fig. 4a, b) [41]. We also observed an increase in the levels of inhibitory GSK-3β phosphorylation at Ser9 that followed the increase in MMP-9 activity (Fig. 4a, b).

**Fig. 5:**
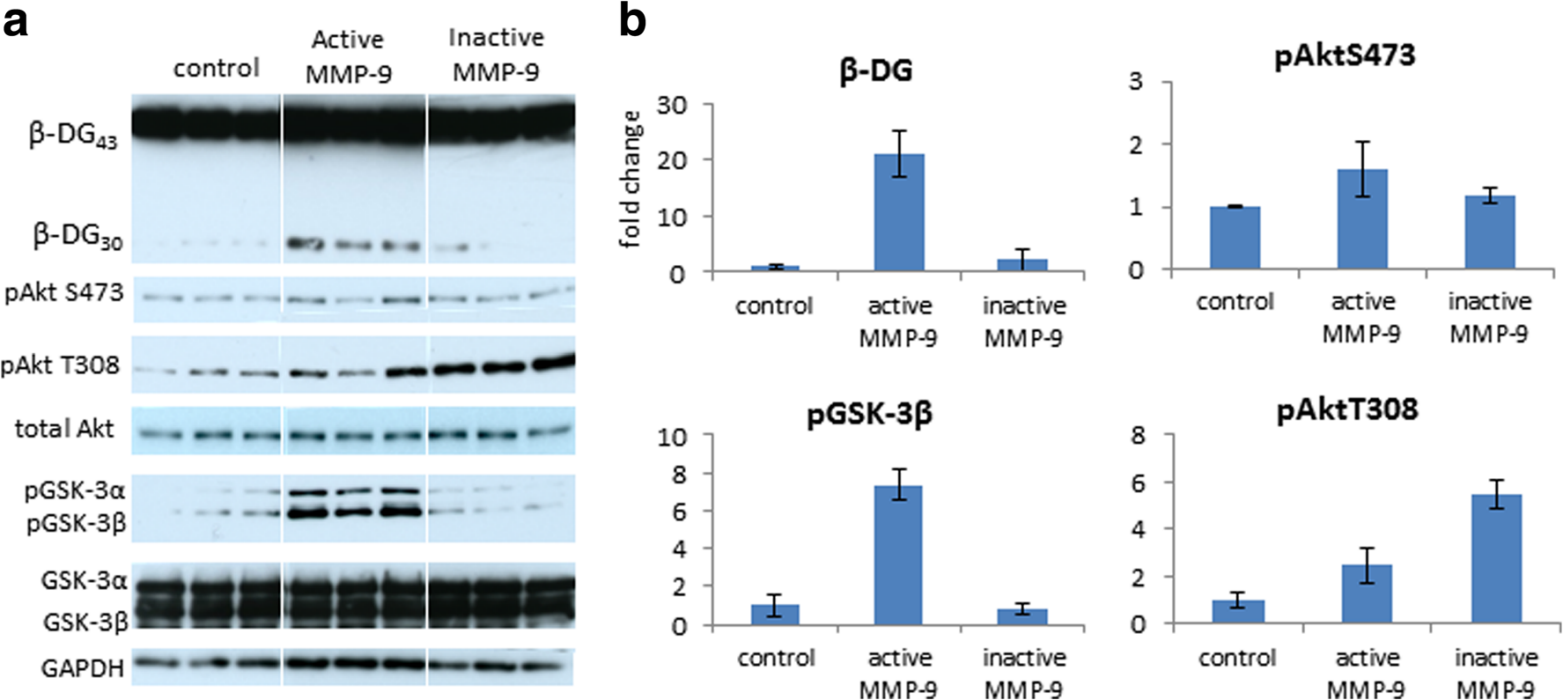
Active and inactive MMP-9 forms differentially regulate GSK-3β and Akt phosphorylations. **a** Hippocampal neurons were incubated for 5 min with 400 ng/ml of recombinant MMP-9 or inactive MMP-9 E402A mutant. **b** Densitometric quantification of β-DG, pAktSer473, pAktThr308, and pGSK-3βSer9. The data are expressed as mean ± SEM. *n* = 3 culture wells for each condition

To confirm that MMP-9 regulated GSK-3β activity, we incubated dissociated hippocampal cultures with recombinant MMP-9 or its inactive mutant MMP-9 E402A as a control. Active MMP-9, but not the inactive mutant MMP-9 E402A, increased the levels of the cleaved β-DG and of phosphorylated GSK-3β at Ser9 and phosphorylated Akt at Ser473 (Figs. 4c, d and 5a, b). Among the factors that regulate GSK-3β, the PI3K/Akt pathway is by far the major signal transducer. Treatment with the PI3K inhibitor wortmannin prevented exogenous MMP-9-induced GSK-3β phosphorylation (Fig. 4c, d), demonstrating that extracellular MMP-9 induced signaling to GSK-3β. Surprisingly, wortmannin alone increased levels of 30-kDa form of β-DG (Fig. 4c, d). Wortmannin, however, did not affect exogenous MMP-9-induced β-DG cleavage (Fig. 4c, d).

## Discussion

Here, we studied the direct role of the GSK-3β isozyme in dendritic spine morphology that is fundamentally and translationally important and in need of in depth examination. We analyzed dendritic spines in the dentate gyrus because we observed previously that GSK-3β affects the volume of the dentate gyrus and related functional aspects such as species-typical behavior [55]. Furthermore, this region is essential in the trisynaptic circuit that processes information from the entorhinal cortex to the CA3 region of the hippocampus [56]. In the present study, we found that an imbalance of GSK-3β activity affects the morphology of dendritic spines bi-directionally. Increasing GSK-3β activity resulted in an elongation of spines, shifting the spine population toward the thin type. Conversely, reducing GSK-3β activity either genetically or pharmacologically resulted in a shortening of spines, shifting their population toward the stubby type. These shifts occurred at the expense of the mushroom spine type, suggesting that GSK-3β can actively switch the balance of dendritic spines toward less mature populations. Likewise, in a different model, GSK-3β deficiency produced similar changes in dendritic spine populations in the CA1 region of the hippocampus [27]. On the other hand, higher neuronal GSK-3β activity decreased postsynaptic density (PSD) in hippocampal granule neurons, indicating less mature spines [28]. Our current results and previous studies demonstrate that GSK-3β activity regulates the morphology of dendritic spines in the dentate gyrus and CA regions of the hippocampus.

### Relation of GSK-3β to Spine Morphology and MMP-9

MMP-9 is a protease that is secreted at excitatory synapses upon enhanced synaptic activity, allowing it to cleave CAMs and thereby reshape synaptic connections and morphology [57–59]. Our results suggest that active GSK-3β promotes the secretion of MMP-9 in response to neuronal excitation, and MMP-9 in turn influences intracellular signaling pathways that involve GSK-3β. MMP-9 was previously shown to activate extracellular signal-regulated kinase 1/2 and Akt in Schwann cells, regardless of its activity (i.e., by hemopexin domain binding to LRP1 receptor) [60]. Our current results showed that the enzymatic activity of MMP-9 is important for initiating the Akt-GSK-3 cascade in neurons. This is supported by our findings of differential Akt phosphorylation at Ser473 and Thr308 upon application of active and inactive MMP-9 forms. Inactive MMP-9 induced only Thr308 phosphorylation suggesting that Akt was not fully active in this condition. Only upon the sequential phosphorylation of Thr308 and Ser473, Akt achieves full activity [61]. Here, PI3K inhibitor wortmannin efficiently blocked active MMP-9-induced Akt and GSK-3β phosphorylations. Interestingly, p110δ PI3K isoform is required for membrane localization of β-DG [62] which may explain the observed increased β-DG cleavage by wortmannin. Whether synaptic MMP-9 substrates, such as β-DG [41] or nectin-3 [63], are involved in signal transduction following application of active MMP-9, remains to be elucidated. Nevertheless, the physiological role for MMP-9 may involve keeping GSK-3 inactive upon neuronal stimulation. In contrast, in brain pathology, GSK-3 overactivation may drive aberrant dendritic spine pathology via activated MMP-9.

Unexpectedly, mice lacking neuronal GSK-3β were hyper-sensitive to PTZ, and their acute death prevented an analysis of MMP-9 activity upon neuronal excitation. MMP-9 basal activity was not affected in either of the GSK-3β-modified mice, possibly because of the low levels of constitutive MMP-9 or because its activity is affected in specific subfields of the hippocampus [41, 42, 64, 65].

Consistent with the increase in the activity of MMP-9 in GSK-3β[S9A] mice, the MMP inhibitor normalized dendritic spines in acute brain slices from GSK-3β[S9A] mice. The data show that higher MMP-9 activity in GSK-3β[S9A] mice translates into a structural outcome that is longer and thinner dendritic spines. Because local protein translation occurs on the order of minutes after synaptic activation [66], GSK-3β is proposed to regulate the local synthesis of MMP-9, in line with our observation that the levels of the MMP-9 precursor increased in GSK-3β[S9A] mice that were treated with PTZ. Although this and previous studies did not provide evidence of the involvement of GSK-3 in local protein translation, the activities of other proteins that are involved in controlling this process, such as p70-S6K [67], TSC2/mTOR [68], and eIF4E [69], were shown to be regulated by GSK-3β.

Our results implicate MMP-9 as an effector of GSK-3β-mediated changes in dendritic spines. The effects of the relationship between GSK-3β and MMP-9 on dendritic spines were further evaluated pharmacologically in MMP-9 KO mice and MMP-9 TG rats. Lithium salts inhibit GSK-3, although not specifically, and normalized in MMP-9 TG rats the longer and thinner dendritic spines. That in MMP-9 KO mice the shorter dendritic spines were not affected by lithium salts is explained by the fact that the observed effect was maximal by the MMP-9 deficiency.

### Relation of Spine Morphology to Synaptic Transmission in Health and Disease

The aberrant morphology of dendritic spines on apical dendrites of granule neurons may have important functional consequences, exemplified by GSK-3β[S9A] mice that present impairments in synaptic transmission and hippocampus-dependent cognitive tasks (i.e., inhibitory avoidance and novel object recognition) [15].

The relation of spine morphology to synaptic transmission and their dynamic regulations in health and disease are inferred, but the underlying mechanisms are not well understood. In general, mushroom spines have a larger PSD with a higher content of glutamate receptors and are more sensitive to glutamate, which is typical for mature synapses. In contrast, thin slender spines are associated with no or a small PSD that contains NMDA receptors but no or only a few α-amino-3-hydroxy-5-methyl-4-isoxazolepropionic acid (AMPA) receptors. These spines appear to be transitional and ready for strengthening and stabilization by the addition of AMPA receptors and an enlarged PSD, or alternatively, to shrink and dismantle, both in response to more or less synaptic inputs. Furthermore, LTP makes spines become larger, whereas LTD causes shrinkage of spines [70]. Because GSK-3β is essential for LTD [14], the shifts to thinner spines in GSK-3β[S9A] mice or to stubby spines in GSK3β^n−/−^ mice unlikely reflected physiological processes associated with LTD. Rather, these changes reflect pathological spine alterations, as observed in neurological and neuropsychiatric disorders.

Increased numbers of elongated, thin dendritic spines are a feature of fragile X syndrome (FXS), a disorder that is characterized by mental retardation [71]. Conversely, a reduction of the number of dendritic spines is evident in neurodegenerative diseases including Alzheimer’s disease, whereas in psychiatric diseases such as schizophrenia and depression alternations in spine morphology in either direction are evident [72].

In FXS, the higher incidence of thin spines is explained by an increase in the activity of MMP-9, which is known to cause the elongation and thinning of dendritic spines [35, 45, 46, 73]. Furthermore, the fragile X mental retardation protein (FMRP) KO mouse model of FXS exhibits increased levels of GSK-3β, whereas mice with active GSK-3α/β isozymes share some autism-related features with FMRP KO mice [74]. GSK-3β was previously reported to downregulate FXR1P, a protein that belongs to a small family of RNA binding proteins that also includes FMRP [75]. Because FMRP KO mice have higher MMP-9 levels [76], we hypothesize that longer and thinner dendritic spines in GSK-3β[S9A] mice are caused by the dysregulation of MMP-9 activity. Indeed, higher GSK-3β[S9A] activity potentiated MMP-9 but not MMP-2 in the hippocampus in PTZ-treated mice.

Altogether, our results demonstrate that the dysregulation of GSK-3β activity in either direction has dramatic consequences on dendritic spine morphology that are reminiscent of dendritic spine alternations that are observed in different neurological and neuropsychiatric disorders. Furthermore, our results offer new insights into the possible synaptic mechanisms of these disorders and indicate potential levels of therapeutic interventions.
